# Behavioral Consequences of Delta-Opioid Receptor Activation in the Periaqueductal Gray of Morphine Tolerant Rats

**DOI:** 10.1155/2009/516328

**Published:** 2009-02-25

**Authors:** Michael M. Morgan, Michelle D. Ashley, Susan L. Ingram, MacDonald J. Christie

**Affiliations:** ^1^Department of Psychology, Washington State University Vancouver, 14204 NE Salmon Creek Avenue, Vancouver, WA 98686, USA; ^2^Brain and Mind Research Institute, The University of Sydney, NSW 2006, Australia

## Abstract

Chronic morphine administration shifts delta-opioid receptors (DORs) from the cytoplasm to the plasma membrane. Given that microinjection of morphine into the PAG produces antinociception, it is hypothesized that the movement of DORs to the membrane will allow antinociception to the DOR agonist deltorphin II as a way to compensate for morphine tolerance. Tolerance was induced by twice daily injections of morphine (5, 10, or 20 mg/kg, subcutaneous) for 3.5 days. Microinjection of deltorphin into the vPAG 6 hours after the last morphine injection produced a mild antinociception that did not vary in a consistent manner across morphine pretreatment doses or nociceptive tests. In contrast, deltorphin caused a decrease in activity in morphine tolerant rats that was associated with lying in the cage. The decrease in activity and change in behavior indicate that chronic morphine administration alters DORs in the vPAG. However, activation of these receptors does not appear to compensate for the decrease in antinociception caused by morphine tolerance.

## 1. Introduction

Opioid receptors in the periaqueductal gray (PAG) contribute to a wide range of behaviors. These include nociceptive modulation, cardiovascular regulation, thermoregulation, and locomotor activity [1–5]. Although mu-opioid receptors (MOR) are known to contribute to PAG mediated antinociception [6, 7], less is known about the contribution of delta-opioid receptors (DORs). Although antinociception has been produced by the administration of DOR agonists into the PAG, these effects are mild compared to the antinociception produced by MOR agonists [8–10].

DORs are particularly interesting because the expression of these receptors is surprisingly dynamic. Chronic treatment with morphine causes the spinal density of DORs to shift from the cytoplasm to the plasma membrane [11, 12]. A similar shift in DORs from the cytoplasm [13] to the plasma membrane appears to occur in the PAG. Swim stress causes an increase in DOR density in the plasma membrane of PAG neurons [14]. *In vitro* recordings show that DOR agonists do not alter GABAergic synaptic transmission in PAG neurons from drug-naive animals [15–17], but inhibit GABAergic IPSCs in mice treated chronically with morphine [18].

The behavioral significance of enhanced DOR expression in the PAG has not been characterized. The increased expression of DOR in the PAG of morphine tolerant rats could be a compensatory mechanism for the loss of antinociception at the mu-opioid receptor. Increased expression of DORs in the spinal cord has been shown to enhance the antinociceptive effect of intrathecal administration of the DOR agonist deltorphin II [19].

The objective of the present study was to determine the behavioral consequences of activating DORs in the PAG following induction of morphine tolerance. Given the widespread effects mediated by the PAG, mobilization of DORs to the plasma membrane could contribute to a wide range of behaviors. The enhanced antinociceptive effects of DOR agonists at the spinal level [19] suggest that the administration of DOR agonists into the vPAG of morphine tolerant rats will produce antinociception. This hypothesis will be tested by examining the antinociceptive and locomotor effects of microinjecting the DOR agonist deltorphin into the vPAG of rats made tolerant to morphine.

## 2. Methods

Male Sprague-Dawley rats (240–360 g) were anesthetized with pentobarbital and implanted with a guide cannula aimed at the ventrolateral PAG using stereotaxic techniques (from lambda: AP = +1.2 mm, ML = 0.6 mm, and DV = −4.6 mm). The guide cannula was 9 mm long and affixed to two screws in the skull with dental cement. Rats were handled daily for one week following surgery. All injections and testing were conducted during the dark phase of a 12-hour light/dark cycle in a dimly illuminated room. Experiments were conducted in accordance with the National Institutes of Health Guide for the Care and Use of Laboratory animals. Efforts were made to minimize the number and potential suffering of the experimental subjects.

### 2.1. Materials

Nociception was assessed using the hot plate, tail withdrawal, and formalin tests. The hot-plate test (IITC, Woodland Hills, Calif, USA) consisted of measuring the latency for a rat to lick a hind paw when placed on a 52°C plate. Tail withdrawal measured the latency to move the tail when placed in 52°C water. The formalin test consisted of rating pain behavior on a 0–3 scale following injection of formalin (2% in 50 ul) into the plantar surface of the hind paw [20]. The values on this scale are 0 = normal behavior; 1 = paw touches the ground without bearing weight; 2 = paw does not touch the ground; 3 = paw is above the ground and licked.

### 2.2. Microinjection Procedure

Four days after surgery, an injection cannula was inserted through the guide cannula, but no drug was injected. This process habituates rats to the injection procedure and diminishes behavioral effects produced by cell damage on the test day. Testing began one week following surgery, deltorphin II (1 *μ*g/0.5 *μ*l) or saline was microinjected into the vPAG. An 11 mm injection cannula was inserted into the guide cannula while the rat was gently restrained by hand. The injection cannula extended 2 mm beyond the end of the guide cannula. Drugs were injected at a rate of 0.1 ul/10 s. The injection cannula remained in place an additional 20 seconds to minimize drug flow up the cannula track. The stylet was reinserted into the guide cannula and the rat was returned to its home cage.

**Experiment 1. Repeated Morphine Injections**

The objective of this experiment was to determine the behavioral effects of microinjecting deltorphin into the vPAG in rats made tolerant to repeated subcutaneous injections of morphine. Morphine (5, 10, or 20 mg/kg) or saline (1 ml/kg) was administered twice a day (at 9:30 and 15:00) for 3.5 days. Nociception was assessed with the hot plate and tail flick tests 30 minutes after the injection on trials 1 and 7, but not after injections on trials 2–6. This procedure limits changes in nociception from repeated testing [21, 22]. Six hours after the last subcutaneous injection, all rats were injected with deltorphin (1 *μ*g/0.5 *μ*l) into the vPAG. Nociception was assessed using the hot-plate test 20 minutes later. A subset of these rats was tested again on the hot plate 50 minutes after deltorphin administration (*N* = 9, 5, 10, and 8 for groups tested with saline, 5, 10, and 20 mg/kg of morphine, resp.). Nociception was assessed using the formalin test in the other rats (*N* = 8, 6, and 9 for groups tested with saline, 10, and 20 mg/kg of morphine, resp.).

Locomotor activity was assessed for 30 minutes beginning immediately after the 20 minutes hot-plate test. Activity was assessed by placing the rat into a chamber (25.1 × 47 cm) with 7 photobeams spaced 5.1 cm apart (San Diego Instruments, San Diego, Calif, USA). The average number of photobeams disrupted each minute was measured and averaged over 10 minute intervals for 30 minutes. The behavior of the rat was examined every 5 minutes during the locomotor test in an attempt to determine the reason for the changes in locomotion (e.g., grooming, sleeping, and freezing). Normal behavior was defined as walking, sniffing, and grooming.

**Experiment 2. Continuous Morphine Administration**

Rats were surgically implanted with a guide cannula aimed at the vPAG as described in Experiment 1. One week later, tolerance was induced by implanting two 75 mg morphine pellets under the skin of the upper back while rats were briefly anesthetized with halothane. Control rats were implanted with two placebo pellets. Nociception was assessed using the hot-plate test 2 hours following pellet implantation.

Rats were returned to the test room 3 days after pellet implantation and allowed to habituate for 30 minutes. Nociception was assessed at the end of this period using the hot-plate test to determine whether tolerance had developed. Following this baseline test, both morphine and placebo-treated rats were injected with deltorphin (1 ug/0.5 ul) into the vPAG. Rats were returned to their cage immediately following the injection. Nociception was assessed using the hot-plate test 30 and 60 minutes after the deltorphin microinjection.

### 2.3. Histology

Following testing, rats were given an overdose of halothane (Sigma, St. Louis, Mo, USA). The microinjection site was marked by injecting cresyl violet (0.2 *μ*l) into the PAG. The brain was removed, placed in formalin (10%), sectioned coronally (50 *μ*m), and viewed under a microscope to localize the injection site [23]. Only rats with injection sites in or immediately adjacent to the vPAG were included in data analysis.

### 2.4. Data Analysis

The effects of morphine pretreatment were compared to saline or placebo-treated controls using a *t*-test or analysis of variance. The Bonferroni and Tukey tests were used for post hoc comparisons. Statistical significance was defined as a probability of less than .05.

## 3. Results

**Experiment 1. Repeated Morphine Injections**

**Figure 1:**
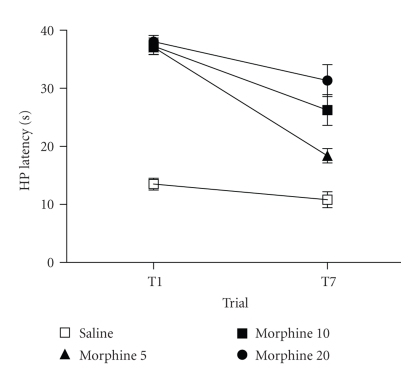
Tolerance to antinociception develops with repeated injections of morphine. The hot-plate latency following morphine administration on trial 7 was significantly less than on trial 1 for the morphine-treated groups (F(1, 39) = 31.38, *P* < .01). Antinociception was still evident with the administration of large doses of morphine (10 and 20 mg/kg), but not as pronounced as on trial 1. Large sample sizes were used for most groups (*N* = 19, 5, 19, and 17 for rats pretreated with saline and morphine at 5, 10, and 20 mg/kg, resp.) because subsets of these rats were subsequently tested using either the formalin test or repeated hot-plate tests.

Systemic administration of high doses of morphine (5, 10, and 20 mg/kg) produced maximal antinociception on trial 1 (see Figure 1). A significant decrease in antinociception was evident with repeated administration from trial 1 to 7 (F(1,56) = 53.446, *P* < .05). The magnitude of the decrease in antinociception was dose dependent (F(3,56) = 5.536, *P* < .05). That is, the lowest dose (5 mg/kg) produced the least antinociception and the highest dose (20 mg/kg) produced the greatest antinociception in morphine tolerant rats.

**Figure 2:**
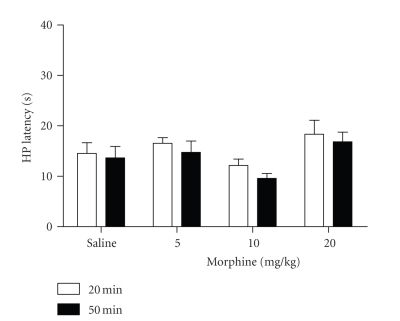
Changes in nociception following microinjection of deltorphin into the vPAG. Nociception was assessed 20 and 50 minutes after deltorphin microinjection into the vPAG of rats made tolerant to morphine. Pretreatment with morphine produced a slight decrease in hot-plate latency in rats made tolerant to 10 mg/kg of morphine compared to the slight increase in rats made tolerant to 20 mg/kg of morphine, although neither of these changes differed significantly from the effects of deltorphin administration in saline-pretreated controls. All rats were tested on the hot plate 20 minutes after deltorphin microinjection (see sample sizes in the caption of Figure 1), but only a subset was tested at 50 minutes for the saline (*N* = 9), 5 (*N* = 5), 10 (*N* = 10), and 20 (*N* = 8) mg/kg morphine groups.

Microinjection of deltorphin into the vPAG of morphine naive rats produced a slight increase in hot-plate latency. Rats pretreated with saline showed a significant increase in hot-plate latency following microinjection of deltorphin from 10.8 (see trial 7 in Figure 1) to 14.5 seconds (20-minute test in Figure 2) (one-tailed *t*(18) = 1.925, *P* < .05).

The effect of microinjecting deltorphin into the vPAG of morphine tolerant rats varied with the pretreatment dose (see Figure 2). A 2 × 2 ANOVA revealed a significant difference in hot-plate latency between the pretreatment groups (F(3,84) = 3.203, *P* < .05), but no difference in hot-plate latency between the 20- and 50-minute tests (F(1,84) = 0.866; *P* > .05). The difference between groups was caused by a slight increase in the hot-plate latency of rats pretreated with 20 mg/kg of morphine and a slight decrease in latency in rats pretreated with 10 mg/kg (see Figure 2). Although the difference between these groups was statistically significant (Bonferroni, *t* = 2.420, *P* < .05), neither the 10 mg/kg (*t* = 0.96 and 1.15 for 20 and 50 minutes, resp.) nor the 20 mg/kg (*t* = 1.49 and 0.87 for 20 and 50 minutes, resp.) groups differed from the saline-pretreated group. Moreover, these changes in nociception were quite small compared to the antinociception produced by systemic administration of morphine on trial 7 (26.2 ± 2.7 and 31.3 ± 2.7 seconds following 10 and 20 mg/kg doses of morphine).

A similar difference between pretreatment groups was evident when nociception was assessed with the tail flick test 50 minutes after deltorphin administration (F(2,52) = 8.699, *P* < .05). Microinjection of deltorphin into the PAG of rats pretreated with 20 mg/kg of morphine caused a small, but significant, increase in tail flick latency (4.4 ± 0.2 seconds) compared to rats pretreated with saline (3.5 ± 0.2 seconds) or 10 mg/kg of morphine (3.4 ± 0.2 seconds) (Tukey test, *P* < .05 for both comparisons).

Nociception was assessed using the formalin test in the rats not tested on the hot plate at 50 minutes. Formalin was injected 5 minutes following the 20 minute hot-plate test so the first and second phases [20] could be assessed 25–29 and 40–44 minutes after microinjection of deltorphin. In contrast to the hot-plate test, pain ratings on the formalin test did not differ between the pretreatment groups on either the first (mean ratings = 1.65, 1.60, and 1.73) or the second (mean ratings = 0.40, 0.63, and 0.67) phases (F(2,20) = 0.272, *P* = .76).

**Figure 3:**
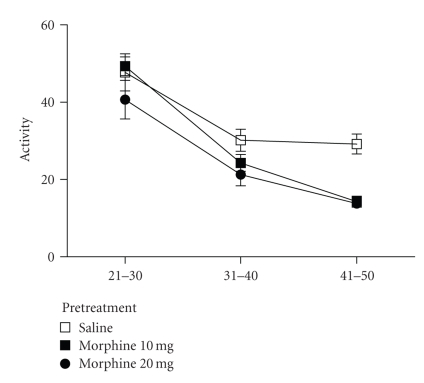
Decrease in locomotion following microinjection of deltorphin into the vPAG. Microinjection of deltorphin (1 *μ*g/0.5 *μ*l) into the vPAG caused a decrease in locomotion in rats pretreated with morphine (*N* = 13 and 9 for rats pretreated with 10 and 20 mg/kg) compared to rats pretreated with saline (*N* = 11). This decrease in activity is caused by a change in normal behavior to lying and crouching in the cage following deltorphin administration (see Table 1).

Locomotor activity was assessed during the 30 minutes following the 20 minute hot-plate test in the subset of rats not injected with formalin. Microinjection of deltorphin into the vPAG caused a significant decrease in activity in the morphine compared to the saline-pretreated rats (F(2,29) = 6.84, *P* < .05). This decrease was similar in rats pretreated with 10 and 20 mg/kg of morphine (see Figure 3). The greatest decrease in activity was evident at the latest time period (41–50 minutes) for both pretreatment groups.

The decrease in activity was accompanied by a change in behavior in which rats tended to crouch or lie down in the activity chamber. An increase in the number of rats crouching or lying increased in rats pretreated with 10 and 20 mg/kg of morphine beginning approximately 25 minutes after the deltorphin microinjection (see Table 1). This shift in the number of rats displaying normal exploratory behavior to crouching and lying increased further during the last 15 minutes of the test (40–54 minutes after the deltorphin microinjection).

**Experiment 2. Continuous Morphine Administration**

**Figure 4:**
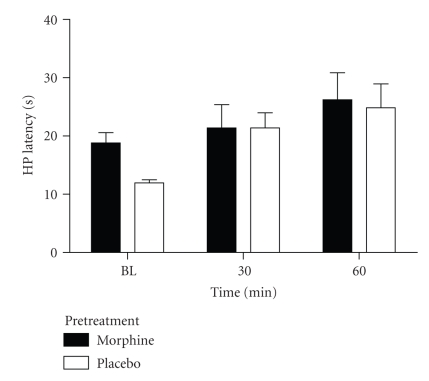
No change in nociception following deltorphin microinjection into the vPAG in rats treated with continuous morphine. Rats implanted with morphine pellets 3 days prior to this test had higher baseline hot-plate latency than rats implanted with placebo pellets indicating that tolerance to the antinociceptive effects was not complete. Microinjection of deltorphin into the vPAG caused an increase in hot-plate latency in rats with placebo pellets (*N* = 5), but did not increase the hot-plate latency for rats with morphine pellets (*N* = 5) above the baseline latency. That is, there was no additional antinociception by injecting deltorphin into the vPAG of rats receiving morphine.

Implantation of morphine pellets produced an increase in hot-plate latency compared to placebo-treated rats when assessed at 1 hour (36.1 ± 3.9 versus 20.5 ± 2.4 seconds; *t*(8) = 3.865, *P* < .05). Tolerance to the antinociceptive effect of morphine was evident by day 4 as indicated by a decrease in hot-plate latency to 18.8 ± 1.8 seconds. Microinjection of deltorphin into the PAG caused a modest, but significant, increase in hot-plate latency compared to baseline latency (F(2,16) = 5.806, *P* < .05). Although the baseline hot-plate latency for rats treated with morphine pellets was greater than in placebo-treated rats (see Figure 4), this difference did not reach statistical significance using the Bonferroni test (*t* = 1.482 nanoseconds). However, microinjection of deltorphin into the vPAG of placebo-treated rats caused a significant increase in hot-plate latency at the 60-minute time point compared to the baseline (Bonferroni, *t* = 3.044. *P* < .05). There was no difference in mean hot-plate latency following deltorphin administration between morphine and placebo-treated rats (F(1,8) = 0.791, *P* > .05), suggesting that deltorphin administration did not increase antinociception beyond what was already present in rats with morphine pellets.

## 4. Discussion

The present data demonstrate that repeated morphine administration alters the response of vPAG neurons to the DOR agonist deltorphin. Although PAG neurons contribute to a wide range of behaviors [24], the change in response to deltorphin microinjection was specific to locomotor activity. Microinjection of deltorphin into the vPAG produced a consistent decrease in activity in rats pretreated with morphine. This decrease in activity was caused by a drastic change in behavior from exploring and grooming to crouching and lying along the edge of the cage. In contrast, microinjection of deltorphin produced a mild antinociception that was not altered in a consistent manner by prior morphine administration.

MOR agonists such as morphine are the most effective treatment for pain. The descending modulatory system that runs from the PAG to rostral ventromedial medulla (RVM) to spinal dorsal horn plays an important role in the antinociceptive effects of both MOR and DOR agonists [9, 25–28]. The antinociception produced by microinjection of DOR agonists into the PAG is weak compared to morphine administration [9, 10]. However, DORs are located in the PAG [13] and these receptors appear to move from the cytoplasm to the plasma membrane following stress [14]. The density of DORs on the membrane has also been shown to increase in spinal neurons following chronic exposure to morphine [11, 12].

The present data show that microinjection of deltorphin into the vPAG had modest effects on nociception. Rats pretreated with 10 mg/kg of morphine showed a slight hyperalgesia compared to saline-pretreated rats injected with deltorphin into the vPAG. This effect seems to be dose and test dependent. Rats pretreated with 20 mg/kg of morphine showed a slight increase in hot-plate latency following deltorphin microinjection. No changes in nociception were evident on the formalin test. The lack of effect of deltorphin in modulating nociception is surprising given that spinal administration of deltorphin following chronic morphine administration produces antinociception [11].

The lack of a consistent change in nociception following deltorphin administration could be caused by an inability of the morphine administration procedure to mobilize DORs to the plasma membrane. Although possible, this explanation seems unlikely given that a decrease in activity was produced by microinjection of deltorphin into the PAG. Moreover, we used two different procedures to induce tolerance (repeated injections and continuous administration) that closely match previous studies reporting changes in DORs [11, 12, 18]. Finally, mobilization of DORs to the plasma membrane is associated with morphine tolerance and the rats in the present study showed clear signs of tolerance to the antinociceptive effects of morphine.

*In vitro* electrophysiological recordings reveal DOR-mediated inhibition of GABAergic IPSCs in tissue from mice pretreated with morphine [18], but no effect of DOR agonists in PAG slices from animals that have not been exposed to morphine [15–17]. This inhibition of GABA input is similar to the effect produced by administration of MOR agonists into the PAG [29]. These data suggest that microinjection of DOR agonists into the PAG of morphine tolerant rats should produce antinociception. However, the present data show no consistent antinociceptive effect following deltorphin microinjection into the PAG of morphine tolerant rats.

In contrast, microinjection of deltorphin into the vPAG of morphine pretreated-rats caused a clear and consistent decrease in locomotor activity. Acute administration of morphine into the vPAG also produces a decrease in activity [4]. Thus, it appears that DORs compensate for the locomotor, but not the antinociceptive effects associated with morphine tolerance in the vPAG. The immobility produced by morphine microinjection into the vPAG appears to be part of a defensive freezing response [30–33]. However, the decrease in activity produced by microinjection of deltorphin into the vPAG reported here does not appear to be caused by fear-induced freezing. Microinjection of deltorphin caused rats to crouch and lay along the edge of the cage as if the rats were ill or dysphoric. This effect appears to be consistent with previous research showing that deep tissue pain sufficient to induce recuperative behavior activates vPAG neurons [34]. Given that stress increases the density of DORs on the plasma membrane [14], activation of these receptors may contribute to recuperative behavior.

One hypothesis is that the recuperative behavior mediated by the vPAG is part of a coordinated response triggered by severe hemorrhage that includes hypotension. Severe blood loss has been shown to activate neurons in the vPAG [35], and inactivation of the vPAG [2, 36] or microinjection of the DOR antagonist naltrindole into the PAG [37] blocks the hypotension produced by hemorrhage. Future studies are needed to determine whether activation of DORs in the PAG alters blood pressure.

## 5. Conclusion

The decrease in locomotor activity caused by microinjection of deltorphin into the vPAG of morphine tolerant rats is consistent with previous data showing that DOR density on the plasma membrane increases following chronic morphine administration. PAG DORs could contribute to morphine tolerance [38–40], behavioral changes related to stress [14], or hypovolemic shock [35, 37], but do not appear to contribute to antinociception. That is microinjection of deltorphin into the PAG did not produce antinociception regardless of how tolerance was induced (repeated injections or continuous administration), test used to assess nociception (hot plate, tail flick, and formalin tests), or test times (20 and 50 minutes).

## Figures and Tables

**Table 1:** Percentage of rats showing normal and abnormal behavior following deltorphin microinjection into the vPAG in morphine-pretreated rats.

Crouching/lying		
Pretreatment	25–39 minutes	40–54 minutes
Saline	0% (0/6)	0% (0/6)
Morphine 10 mg	20% (1/5)	67% (6/9)
Morphine 20 mg	36% (4/11)	40% (6/15)

Note: sample sizes are shown in parentheses.
